# Analyses of the Effects of Arginine, Nicotine, Serotype and Collagen-Binding Proteins on Biofilm Development by 33 Strains of *Streptococcus mutans*

**DOI:** 10.3389/froh.2021.764784

**Published:** 2021-11-25

**Authors:** Dawn R. Wagenknecht, Richard L. Gregory

**Affiliations:** Department of Biomedical Science and Comprehensive Care, Indiana University School of Dentistry, Indianapolis, IN, United States

**Keywords:** *cbm* gene, *cnm* gene, arginine, nicotine, *Streptococcus mutans*, biofilm, serotype *k*

## Abstract

*Streptococcus mutans* serotype *k* strains comprise <3% of oral isolates of *S. mutans* but are prominent in diseased cardiovascular (CV) tissue. Collagen binding protein (CBP) genes, *cbm* and *cnm*, are prevalent in serotype *k* strains and are associated with endothelial cell invasion. Nicotine increases biofilm formation by serotype *c* strains of *S. mutans*, but its effects on serotype *k* strains and strains with CBP are unknown. Saliva contains arginine which alters certain properties of the extracellular polysaccharides (EPS) in *S. mutans* biofilm. We examined whether nicotine and arginine affect sucrose-induced biofilm of *S. mutans* serotypes *k* (*n* = 23) and *c* (*n* = 10) strains with and without CBP genes. Biofilm mass, metabolism, bacterial proliferation, and EPS production were assessed. Nicotine increased biomass and metabolic activity (*p* < 0.0001); arginine alone had no effect. The presence of a CBP gene (either *cbm* or *cnm*) had a significant effect on biofilm production, but serotype did not. Nicotine increased bacterial proliferation and the effect was greater in CBP + strains compared to strains lacking CBP genes. Addition of arginine with nicotine decreased both bacterial mass and EPS compared to biofilm grown in nicotine alone. EPS production was greater in *cnm* + than *cbm* + strains (*p* < 0.0001). Given the findings of *S. mutans* in diseased CV tissue, a nicotine induced increase in biofilm production by CBP + strains may be a key link between tobacco use and CV diseases.

## Introduction

*Streptococcus mutans*, a gram-positive oral bacterium key to development of dental caries is classified by serotype based upon the composition and structure of cell wall polysaccharides. Serotypes c, e and *f* have glucose side chains on a rhamnose backbone [1]. In contrast, for strains of serotype *k* there is a dearth of glucose side chains on the rhamnose backbone [2]. Serotype *c* strains of *S. mutans* are most prevalent. Strains of serotypes *f* and *k* comprise <5% of the *S. mutans* isolated from the oral cavities of healthy individuals but these serotypes are significantly increased in oral isolates from patients with cardiovascular disease (CVD) and infective endocarditis [1, 3, 4]. Diseased heart valves and atherosclerotic plaque tissues harbor a higher prevalence of serotypes *f* and *k S. mutans* strains than observed in the oral isolates of the same subjects [3]. The presence of collagen binding proteins (CBP) Cbm and Cnm has been shown to facilitate *S. mutans* invasion of endothelial cells and heart valve tissues [3, 5–8]. The genes for these CBP, *cbm* and *cnm*, are found predominantly in *S. mutans* serotypes *f* and *k* strains.

Tobacco use is associated with increased risk for CVD and oral diseases, including caries [9]. Nicotine, a bioactive agent in tobacco, has been shown to increase *in vitro* growth and metabolism of *S. mutans* serotypes c, e and *f* strains grown in planktonic and biofilm cultures. Huang et al. found minor enhancement of planktonic growth in seven strains of *S. mutans* upon addition of 1 and 2 mg/ml nicotine [10]. In contrast, biofilm growth of the same seven strains increased in a nicotine dose dependent manner until toxicity was reached at 16 or 32 mg/ml nicotine depending upon the individual strain. Metabolism of established *S. mutans* biofilm increased and the cellular morphology changed from the usual ellipsoid shape to a more spherical shape with increasing nicotine exposure [10]. Confocal laser scanning microscopy of *S. mutans* biofilm revealed both bacterial volume and extracellular polysaccharide (EPS) volume were increased in the presence of nicotine [11]. Nicotine had significant effects on biofilm growth of serotypes c, e and *f* strains of *S. mutans*. The effects of nicotine on serotype *k* strains of *S. mutans* have not been investigated.

The basic amino acid, arginine, is present in whole saliva and in ductal saliva collected from parotid glands [12, 13]. The concentration of arginine in parotid saliva is increased in caries-free compared to caries-susceptible individuals suggesting that arginine may play a role in the control of caries development [13]. Free arginine is also found in the endothelial glycocalyx at the site of *S. mutans* invasion into cardiovascular tissues [14, 15]. *In vitro* studies have demonstrated that the adhesive and structural properties of *S. mutans* biofilm are altered in the presence of arginine compared to biofilm grown without supplemental arginine. By using atomic force microscopy, Sharma et al. demonstrated both adhesion force and rupture length for *S. mutans* biofilms were decreased with addition of arginine in a dose dependent manner leaving the biofilm with a more fragile structure [12]. *In vitro* studies of serotype *c* strains demonstrated arginine increased biomass and decreased EPS compared to *S. mutans* biofilm cultured in the absence of arginine [16]. Other studies demonstrated arginine causes downregulation of genes encoding virulence factors responsible for attachment, competence development and bacteriocin production [17]. In addition, arginine decreased tolerance to environmental acid and oxidative stresses [17]. The effects of arginine synergize with fluoride to suppress *S. mutans* planktonic and biofilm cultures *in vitro* and induced increased protection against caries lesions in clinical trials of dentifrices [16, 18]. Nicotine caused alterations of *S. mutans* biofilm result in a more abundant biofilm. In contrast, alterations in biofilm in the presence of arginine result in diminished integrity of the biofilm compared to increased biofilm production associated with nicotine. Thus, we investigated the combined effects of the two agents.

The effects of nicotine and arginine on *S. mutans* biofilm have been separately investigated using serotypes c, e and *f* strains of *S. mutans* [10–12, 16, 17]. Further, most of these studies have been carried out with but a few reference strains. Ours is the first study to investigate the combined effects of arginine and nicotine on *S. mutans* biofilm growth and structure using a collection of clinical strains. We report the effects of nicotine and arginine on biofilm from 33 clinical strains of *S. mutans* of known serotype and CBP genotype. There were no significant differences in biofilm production or responses to nicotine and arginine between serotypes *c* and *k* strains. Strains with CBP were more metabolically active and produced significantly more biofilm than strains without CBP. Addition of nicotine resulted in increased biofilm across all strains. In contrast, when arginine was combined with nicotine the quantity of biofilm decreased. Tobacco use and nicotine exposure have been associated with risk for CVD. Inasmuch as nicotine increases *S. mutans* biomass and addition of arginine decreases the biomass, addition of arginine to dentifrices may benefit tobacco users as a counter to the biofilm enhancing effects of nicotine.

## Materials and Methods

### Bacterial Strains and Growth Conditions

The *S. mutans* strains used in this study were kind gifts of Drs. Noel Childers and Stephanie Momeni at University of Alabama School of Dentistry, USA; Kazuhiko Nakano at Osaka University Graduate School of Dentistry, Japan; and Kazuko Takada, Nihon University School of Dentistry at Matsudo, Chiba, Japan. Each strain was serotyped and genotyped by PCR methods by the respective contributors and the genetic type of each strain is listed in Table 1. Frozen stock of each strain was inoculated into tryptic soy broth (TSB; Becton, Dickinson and Co., Sparks, MD, USA) and incubated at 37°C, 5% CO_2_ for 18–48 h, depending on bacterial growth rate, prior to each experiment.

**Table 1:** *Streptococcus mutans* serotype k (*n* = 23) and c (*n* = 10) strains.

**Strain**	**Serotype**	***cbm***	***cnm***	**Source**
106-1-PP3-06-01*	*k*	–	–	S. Momeni, N. Childers^1^
151-1-PP19-07-05*	*k*	–	–	S. Momeni, N. Childers
505-1-PBB-05-06	*k*	–	–	S. Momeni, N. Childers
548-1-PBT-05-04*	*k*	+	–	S. Momeni, N. Childers
573-1-PBS-05-03	*k*	–	–	S. Momeni, N. Childers
578-1-PBB-06-01	*k*	+	–	S. Momeni, N. Childers
608-1-PBB-06-03	*k*	+	–	S. Momeni, N. Childers
AT1	*k*	+	–	K. Nakano^2^
FT1	*k*	–	–	K. Nakano
LJ23	*k*	–	+	K. Nakano
NN2193-1	*k*	+	–	K. Nakano
NN2323M-1	*k*	+	–	K. Nakano
NUM-Smk51	*k*	n/a^3^	n/a	K. Takada^4^
NUM-Smk52	*k*	n/a	n/a	K. Takada
NUM-Smk89	*k*	n/a	n/a	K. Takada
OR22P1	*k*	–	+	K. Nakano
SA31	*k*	+	–	K. Nakano
SA53	*k*	–	+	K. Nakano
TLJ106-1	*k*	–	–	K. Nakano
TLJ11b	*k*	+	–	K. Nakano
TLJ60a	*k*	–	+	K. Nakano
TLJ85d	*k*	+	–	K. Nakano
YT1	*k*	+	–	K. Nakano
107-1-PP3-07-02*	*c*	+	–	S. Momeni, N. Childers
173-1-PP3-06-05	*c*	–	–	S. Momeni, N. Childers
196-1-PP3-07-04	*c*	–	+	S. Momeni, N. Childers
219-1-PP30-07-07*	*c*	–	+	S. Momeni, N. Childers
247-1-PP30-07-03	*c*	+	+	S. Momeni, N. Childers
531-1-PBI-07-06	*c*	+	–	S. Momeni, N. Childers
582-1-PBB-06-02	*c*	+	+	S. Momeni, N. Childers
586-1-PBT-07-02	*c*	+	–	S. Momeni, N. Childers
608-1-PBB-06-01	*c*	+	+	S. Momeni, N. Childers
UA159*	*c*	–	–	ATCC 700610

*^1^University of Alabama School of Dentistry, Birmingham, AL, USA.*

*^2^Osaka University Graduate School of Dentistry, Osaka, Japan.*

*^3^Not available (n/a).*

*^4^Nihon University School of Dentistry at Matsudo, Chiba, Japan.*

*Denotes strains used in CLSM analyses.

### Biofilm Experiments

Experiments were performed in 96-well untreated, polystyrene flat-bottom microtiter plates using TSB with 1% sucrose (TSBS) to promote biofilm formation. Stock solutions of TSBS containing 200 mg/ml arginine (Sigma-Aldrich, St. Louis, MO, USA) in TSBS were adjusted to the pH of TSBS (pH 7.2) prior to sterilization. TSBS was used to dilute the stock arginine-TSBS to the desired concentration. Nicotine (Sigma-Aldrich) was added to TSBS immediately prior to each experiment. Briefly, 100 μl of TSBS without or with double the desired concentration of nicotine and/or arginine was added to quadruplicate wells of a 96-well microtiter plate. Next, 100 μl of overnight *S. mutans* culture (adjusted to 0.500 ± 0.050 OD_595_) diluted 1:50 into TSBS (10^6^ *S. mutans* cells) was added to each well-resulting in the stated final concentrations of nicotine and arginine. Biofilms were grown separately in nicotine and arginine, in the absence of either and in the presence of both and in quadruplicate wells for each treatment. The microtiter plates were incubated for 24 h at 37°C in 5% CO_2_. Each experiment was repeated three times.

### Crystal Violet Assay

In order to assess biofilm mass after 24 h, the biofilm was stained with crystal violet. Briefly, after removing the growth media, each biofilm was washed three times with sterile distilled H_2_O (dH_2_O) and the plates were blotted on absorbent paper prior to 30 min room-temperature incubation with 10% formaldehyde (Sigma-Aldrich). After three additional dH_2_O washes, a 0.5% crystal violet solution (Sigma-Aldrich) was incubated on the biofilm for 30 min followed by additional dH_2_O washes to remove excess crystal violet. Next 2-propanol was added for 60 min to extract the crystal violet prior to reading the optical density of each well at 490 nm on a SpectraMax Plus spectrophotometer (Molecular Devices, Sunnyvale, CA, USA).

### XTT Assay

*S. mutans* cellular metabolism within the biofilm was measured by a 2, 3-bis (2-methoxy-4-nitro-5-sulfophenyl)-5-[(phenylamino) carbonyl]-2H-tetrazolium hydroxide (XTT) reduction assay as previously described [10]. Briefly, the biofilm was washed three times with sterile 0.9% NaCl (Sigma-Aldrich) prior to incubation with 0.2 mg/ml XTT salt (Sigma-Aldrich) in 10% ethanol with 3 μg/ml menadione (Sigma-Aldrich) for 2 h at 37°C in the dark. After incubation, the XTT solution from each well was transferred to the same relative well in a fresh microtiter plate and the optical density at 490 nm was read in a spectrophotometer.

### Confocal Laser Scanning Microscopy

Biofilm was grown directly on 8-chamber cover glasses (Nunc Lab-Tek™II, ThermoFisher Scientific, Waltham, MA, USA) according to the method of Huang, Li et al. [10] by inoculating 18-h planktonic cultures into TSBS containing the appropriate additive and 1.0 μM Alexa Fluor 568 labeled dextran (ThermoFisher Scientific), to label developing EPS. The biofilms were incubated at 37°C in 5% CO_2_ for 24 h then washed 3 times with sterile dH_2_O. Next, bacterial cells within the biofilm were stained with Syto9 (1:5000, ThermoFisher Scientific) for 15 min in the dark at room-temperature before three additional washes with sterile dH_2_O. After the final wash, the biofilms were air-dried then covered with Prolong™ Gold Antifade Mountant (ThermoFisher Scientific). Fluorescent images were obtained using an Olympus FV1000 MPE confocal laser scanning microscope with Olympus FV10-ASW software (Olympus Corp., Center Valley, PA, USA) at the Indiana Center for Biological Microscopy, Indiana University School of Medicine. Full-depth Z-stacks were collected from at least three distinct fields from each cover glass grown biofilm. Up to 7 fields per biofilm were collected when visual inspection through the CLSM revealed visually distinct regions within a biofilm. All Z-stacks from the total depth of each field were processed. The total volumes of EPS (red, Alexa Fluor 568) and bacteria (green, Syto9) in the processed images were analyzed by Imaris Image Analysis Software version 8 (Bitplane, South Windsor, CT, USA) and the volumes of EPS and bacteria were expressed in pixels.

### Statistical Analyses

Each crystal violet and XTT experiment was performed in quadruplicate microtiter wells and independently repeated three times. For the CLSM experiments, a minimum of three separate microscopic fields were collected for each strain and treatment combination. Z-stacks were collected from the full-thickness observed in each field between the first visible fluorescence on the surface of the cover glass to the last visible fluorescence at the top of the biofilm. Basic statistics (mean, SD, etc.) were computed prior to analyses by two-way ANOVA with interaction and random effect for multiple measures using SAS version 9.4 (SAS Institute, Inc., Cary, NC). When non-normality was present, a rank transformation was performed prior to analysis. Data were considered significantly different when the *P*-value was < 0.05.

## Results

### Effects of Arginine and Nicotine on *S. mutans* Biofilm

Details of the of nicotine and arginine dose curves used to optimize the following experiments are provided in the Supplementary Materials and in Supplementary Figures 1, 2.

**Figure 1:**
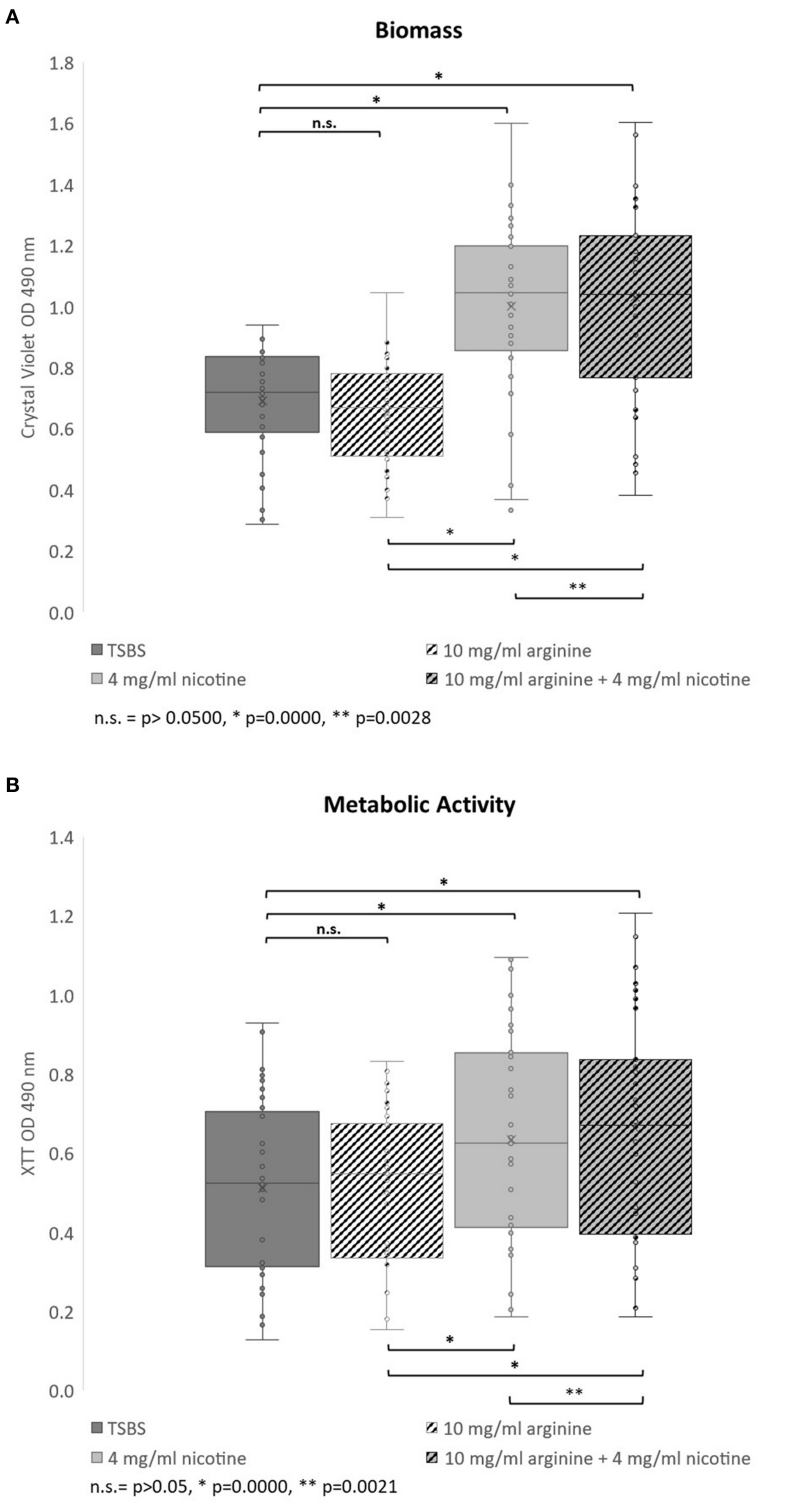
Bar and whisker plots showing the separate and combined effects of arginine and nicotine on *S. mutans* 24 h biofilm growth and metabolic activity. Individual biofilms from all 33 strains were grown for 24 h in TSBS (dark gray), 10 mg/ml arginine (black and white diagonal), 4 mg/ml nicotine (light gray) and 10 mg/ml arginine plus 4 mg/ml nicotine (gray and black diagonal) and **(A)** stained with crystal violet to measure biomass or **(B)** tested for metabolic activity by the XTT assay. Statistical comparisons are shown in the horizontal bars above and below the plots.

The effects of nicotine and arginine, separately and in combination, were assessed in parallel biofilm experiments with 10 strains of serotype *c* and 23 serotype *k* strains. Biofilm grown in TSBS served as the biofilm baseline control for the same strain grown concurrently in 10 mg/ml arginine, 4 mg/ml nicotine, or 10 mg/ml arginine plus 4 mg/ml nicotine. Biofilm of each strain was grown in quadruplicate wells in a microtiter plate and in three separate experiments. Figure 1 summarizes the biofilm produced by all 33 strains in box and whisker plots. Biomass measured by crystal violet staining is shown in panel A and metabolic activity measured by XTT is displayed in panel B. The boxes represent the inter quartile range for quartiles 2 and 3; the median value is shown by the line across the box. The minimum and maximum values are indicated at the ends of the deviation bars or whiskers. Biofilm grown in 10 mg/ml arginine was not different from biofilm grown in TSBS alone. Addition of 4 mg/ml nicotine significantly increased the biomass and metabolic activity of 24 h *S. mutans* biofilm (*p* = 0.0000). Similarly, the combination of nicotine and arginine increased *S. mutans* biofilm mass and metabolic activity compared to nicotine alone (*p* = 0.0028 and 0.0021, respectively).

When the 33 *S. mutans* strains were analyzed by serotype, neither the biomass nor metabolic activity was significantly different between serotypes *c* and *k* for all treatments collectively (*p* = 0.6093 and 0.2650, respectively; data not shown). Nicotine and arginine treatments did not affect the biomass of serotype *c* strains differently than serotype *k* strains (Supplementary Table 1). Biofilms from serotype *k* strains were not significantly different from those produced by serotype *c* strains when analyzed by individual treatment group.

We next asked whether CBP genotype influenced biofilm biomass or metabolic activity. The CBP genotype was known for 30 of the 33 strains tested; 13 strains were *cbm*+/*cnm*-, seven were *cbm*-/*cnm*+, three had both genes and seven strains had neither gene. For biofilm grown in TSBS *cbm*-/*cnm*+ strains produced significantly increased biomass compared to strains lacking CBP genes, *cbm*-/*cnm*- (*p* = 0.0032, Table 2). In contrast, the *cbm*+/*cnm*- strains exhibited a non-significant increase in biomass compared to *cbm*-/*cnm*- and *cbm*+/*cnm*+ strains. Compared to biofilm cultured in TSBS alone, addition of arginine resulted in significantly decreased biomass for *cbm*+/*cnm*- strains. Conversely, nicotine significantly increased the biofilm mass of *cbm*+/*cnm*-, *cbm*-/*cnm*+ and *cbm*-/*cnm*- stains. No increase in biofilm mass occurred when nicotine was added to strains with both CBP, however, these strains demonstrated increased biofilm mass when arginine was added to the nicotine (*p* = 0.0001).

**Table 2:** The effects of *S. mutans* CBP genotype on biofilm mass measured by crystal violet in response to arginine (10 mg/ml) and nicotine (4 mg/ml).

					**Effects of arginine and nicotine**			**Effects of** ***cbm*** **and** ***cnm***		
**Arginine** **(10 mg/ml)**	**Nicotine** **(4 mg/ml)**	**Genotype (n)**	**OD 490 nm**		**vs.** ***arginine***	**vs.** ***nicotine***	**vs.** ***arginine + nicotine***	**vs.** ***cbm*+/*cnm*+**	**vs.** ***cbm*+/*cnm*-**	**vs.** ***cbm*-/*cnm*+**
			**Mean**	**SD**	***P*-value**	***P*-value**	***P*-value**	***P*-value**	***P*-value**	***P*-value**
		*cbm*-/*cnm*- (7)								
–	–		0.606	0.212	0.4014	0.0000	0.0000	0.6737	0.0666	0.0032
+	–		0.584	0.228		0.0000	0.0000	0.3106	0.3165	0.0070
–	+		0.872	0.388			0.7210	0.1125	0.0160	0.0000
+	+		0.862	0.365				0.9594	0.1026	0.0000
		*cbm*+/*cnm*+ (3)								
–	–		0.626	0.259	0.4596	0.1876	0.0000		0.3535	0.0554
+	–		0.661	0.206		0.2028	0.0000		0.7251	0.2650
–	+		0.688	0.309			0.0001		0.0004	0.0000
+	+		0.837	0.285					0.2379	0.0006
		*cbm*+/*cnm*- (13)								
–	–		0.712	0.233	0.0001	0.0000	0.0000			0.1204
+	–		0.647	0.201		0.0000	0.0000			0.0340
–	+		1.029	0.344			0.0481			0.0167
+	+		1.020	0.440						0.0006
		*cbm*-/*cnm*+ (7)								
–	–		0.777	0.133	0.1199	0.0000	0.0000			
+	–		0.746	0.181		0.0000	0.0000			
–	+		1.190	0.243			0.3527			
+	+		1.284	0.308						

Table 3 displays the metabolic activity data for biofilms grown in the four different TSBS combinations. When grown in TSBS without an additive, the metabolic activity of *cbm*-/*cnm*+ strains was not significantly different from the *cbm*+/*cnm*- strains (*p* = 0.1702). Biofilm of strains with either *cbm* or *cnm*, but not both genes, had increased metabolic activity compared to strains lacking CBP (*p* ≤ 0.0002) and strains with both genes (*p* = 0.0359 and 0.0040, respectively). In general, neither arginine nor nicotine altered these observations. Addition of 4 mg/ml nicotine to TSBS resulted in significantly increased biofilm metabolic activity for all strains compared to TSBS alone (*p* ≤ 0.0008). When arginine and nicotine were combined, the metabolic activity of *cbm*-/*cnm*+ strains increased significantly compared to *cbm*+/*cnm*- strains (*p* = 0.0172). The relative metabolic increase in *cbm*-/*cnm*+ strains was greater when compared to *cbm*-/*cnm*- and *cbm*+/*cnm*+ strains (*p* < 0.0001 and =0.0032, respectively). Addition of arginine to nicotine significantly increased the metabolic activity of *cbm*-/*cnm*+ and *cbm*+/*cnm*+ compared to biofilm grown in nicotine alone (*p* = 0.001 and 0.0023, respectively). No significant increase was seen for *cbm*+/*cnm*- and *cbm*-/*cnm*- strains.

**Table 3:** The effects of *S. mutans* CBP genotype on biofilm metabolic activity in response to arginine (10 mg/ml) and nicotine (4 mg/ml).

					**Effects of arginine and nicotine**			**Effects of** ***cbm*** **and** ***cnm***		
**Arginine** **(10 mg/ml)**	**Nicotine** **(4 mg/ml)**	**Genotype (*n*)**	**OD 490 nm**		**vs.** **arginine**	**vs.** **nicotine**	**vs.** **arginine + nicotine**	**vs.** ***cbm*+/*cnm*+**	**vs.** ***cbm*+/*cnm*-**	**vs.** ***cbm*-/*cnm*+**
			**Mean**	**SD**	***P*-value**	***P*-value**	***P*-value**	***P*-value**	***P*-value**	***P*-value**
		*cbm*-/*cnm*- (7)								
–	–		0.355	0.267	0.2801	0.0008	0.0000	0.5376	0.0002	0.0000
+	–		0.341	0.257		0.0000	0.0000	0.2480	0.0001	0.0000
–	+		0.419	0.340			0.5754	0.1894	0.0000	0.0000
+	+		0.430	0.347				0.0708	0.0000	0.0000
		*cbm*+/*cnm*+ (3)								
–	–		0.419	0.144	0.1775	0.0000	0.0000		0.0359	0.0040
+	–		0.464	0.095		0.0000	0.0000		0.1080	0.0172
–	+		0.517	0.119			0.0023		0.0214	0.0022
+	+		0.572	0.113					0.1518	0.0032
		*cbm*+/*cnm*- (13)								
–	–		0.593	0.232	0.4294	0.0000	0.0000			0.1702
+	–		0.573	0.194		0.0000	0.0000			0.1894
–	+		0.745	0.282			0.9997			0.1742
+	+		0.745	0.285						0.0172
		*cbm*-/*cnm*+ (7)								
–	–		0.654	0.136	0.4162	0.0000	0.0000			
+	–		0.650	0.156		0.0000	0.0000			
–	+		0.814	0.206			0.0001			
+	+		0.914	0.205						

### Confocal Laser Scanning Microscopy

**Figure 2:**
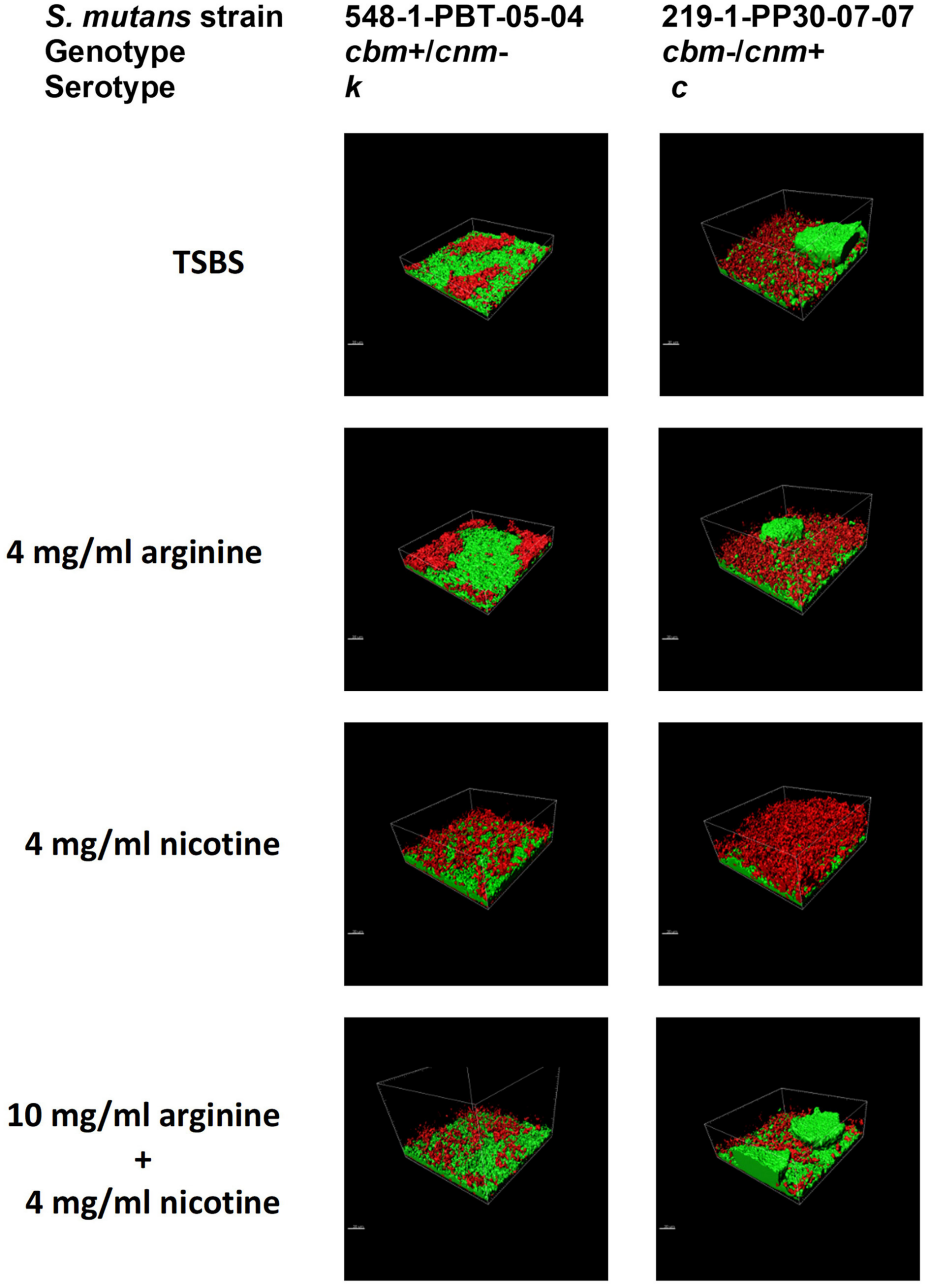
*S. mutans* bacterial cells and EPS from 24 h biofilm measured by CLSM. Alexa Fluor 568 labeled dextran (red) was added at the beginning of biofilm formation to label the EPS as it was synthesized. Arginine and nicotine were added simultaneously with the Alexa Fluor 568 labeled dextran. Prior to imaging the 24 h biofilm, *S. mutans* DNA was labeled with Syto 9 (green). Representative processed Z-stack images from CLSM analyses of biofilm from two selected strains of *S. mutans* grown in TSBS alone, 10 mg/ml arginine, 4 mg/ml nicotine or 10 mg/ml arginine + 4 mg/ml nicotine are shown.

Crystal violet stains the bacterial cells as well as the EPS produced by the *S. mutans* during biofilm formation. By comparison, the XTT assay detects metabolic activity of *S. mutans* and the amount of EPS production is not directly measured in this assay. We therefore used CLSM to separately measure *S. mutans* nucleic acid (Syto9, green) and EPS (Alexa Fluor 568, red) in the biofilm. Images of the full biofilm thickness were analyzed with Imaris Image Analysis Software to create full-depth three-dimensional images of the biofilm to separately quantitate the amount of *S. mutans* and EPS in each field. Six strains representing serotype c (*n* = 3) and *k* (*n* = 3) and three CBP genotypes *cbm*-/*cnm*- (*n* = 3), *cbm*+/*cnm*- (*n* = 2) and *cbm*-/*cnm*+ (*n* = 1) were selected for CLSM analyses (the selected strains are designated by ^*^ in Table 1). Our objective was to study biofilm produced by *S. mutans* strains without CBP genes or one of the genes, therefore, strains with both *cbm* and *cnm* were not included in this experiment. Representative images of single CLSM fields are shown in Figure 2. In most images, *S. mutans* and EPS were found interspersed throughout the biofilm. Images of strain 219-1-PP30-07-07 contained full thickness towers of *S. mutans* with little to no EPS visible within the towers. Analyses of the green pixels in each image revealed that neither serotype nor genotype had a significant effect on the amount of *S. mutans* in the 24 h biofilms (*p* = 0.8325 and 0.3391, respectively; data not shown). In contrast, the effects of serotype and genotype on EPS production were significant (*p* = 0.0145 and < 0.0001, respectively). As shown in Table 4, the *cbm*-/*cnm*+ strain produced significantly more EPS than the *cbm*+/*cnm*- and *cbm*-/*cnm*- strains (*p* < 0.0001 and < 0.0001). The serotype c strains produced significantly more EPS than the serotype *k* strains (*p* = 0.0145), however, this observation is confounded because the single *cnm* positive strain studied by CLSM is serotype c. The amount of EPS produced by the strains lacking the *cnm* gene was not different for strains with or without the *cbm* gene (*p* = 0.9396).

**Table 4:** Pairwise comparisons of the effects of genotype on *S. mutans*, EPS and EPS:*S. mutans* ratio in biofilm as measured by CLSM in pixels.

		**Direction** **of effect**		***P*-value**
***S. mutans***				
	*cbm*+/*cnm*-		*cbm*-/*cnm*+	n.s.
	*cbm*+/*cnm*-		*cbm*-/*cnm*-	n.s.
	*cbm*-/*cnm*+		*cbm*-/*cnm*-	n.s.
**EPS**				
	*cbm*+/*cnm*-	<	*cbm*-/*cnm*+	<0.0001
	*cbm*+/*cnm*-		*cbm*-/*cnm*-	0.9396
	*cbm*-/*cnm*+	>	*cbm*-/*cnm*-	<0.0001
**EPS:** ***S. mutans***				
	*cbm*+/*cnm*-	<	*cbm*-/*cnm*+	0.0001
	*cbm*+/*cnm*-		*cbm*-/*cnm*-	0.4316
	*cbm*-/*cnm*+	>	*cbm*-/*cnm*-	<0.0001

*n.s. = not significant*.

**Figure 3:**
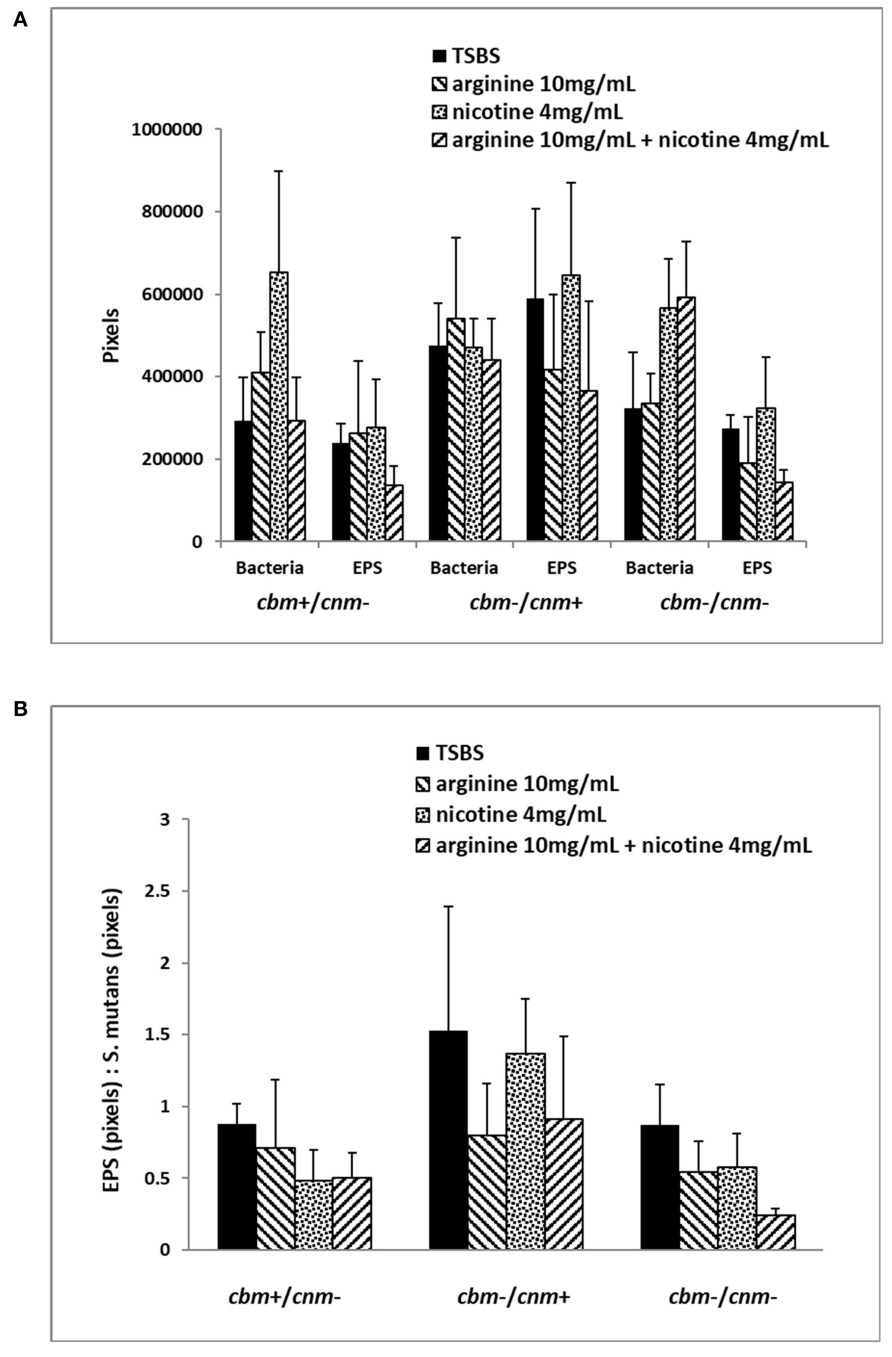
Six selected strains of *S. mutans* bacterial cells representing *cbm*+/*cnm*-, *cbm*-/*cnm*+ and *cbm*-/*cnm*- genotypes were used for comparison of bacterial cell mass and EPS in 24 h biofilm as measured by CLSM. The effects of 10 mg/ml arginine, 4 mg/ml nicotine and the combination on *S. mutans* biofilm were quantified by CLSM. Alexa Fluor 568 labeled dextran was added at the beginning of biofilm formation to label the EPS as it was synthesized. Arginine and nicotine were added simultaneously with the Alexa Fluor 568 labeled dextran. Prior to imaging the 24 h biofilm, *S. mutans* DNA was labeled with Syto 9. Biofilm grown in TSBS alone (black) was compared to biofilm grown with 10 mg/ml arginine (black and white diagonal), 4 mg/ml nicotine (gray) and 10 mg/ml arginine plus 4 mg/ml nicotine (black and gray diagonal). Panel **(A)** shows the total pixels representing bacterial cell mass and EPS for each genotype of *S. mutans* tested by CLSM. Panel **(B)** compares the ratio of EPS to *S. mutans* bacterial cells for each genotype and treatment.

The CLSM analyses revealed that treatment of the biofilm with arginine and nicotine had significant effects on both *S. mutans* (*p* = 0.0069) and EPS production (*p* = 0.0032). Addition of 4 mg/ml nicotine to TSBS resulted in increased biofilm volume of *S. mutans* compared to biofilm grown in TSBS alone and arginine supplemented media for the *cbm*+/*cnm*- and *cbm*-/*cnm*- strains (Figure 3A). Addition of arginine to nicotine decreased *S. mutans* bacterial cell volume compared to nicotine alone (*p* = 0.0404, Table 5). Further, the bacterial cell mass grown in arginine and nicotine was not significantly different from biofilm grown in TSBS or arginine without nicotine (*p* = 0.1748 and 0.8213, respectively). Similarly, nicotine increased EPS production but addition of arginine to nicotine in the growth media resulted in decreased EPS production by *S. mutans* (*p* = 0.0006). To assess the combined effects on *S. mutans* volume and EPS production, the ratio of EPS to *S. mutans* was evaluated for each genotype and treatment (Figure 3B). The ratio of EPS to *S. mutans* volume was increased for the strain with the *cnm* gene compared to strains with the *cbm* gene and the strains lacking both genes. Addition of nicotine, arginine or the combination decreased the EPS:*S. mutans* volume ratio significantly indicating that the bacterial volume increased disproportionally to EPS production in the presence of nicotine and/or arginine.

**Table 5:** Pairwise comparisons of the effects of treatment on *S. mutans*, EPS and EPS:*S. mutans* ratio in biofilm as measured by CLSM in pixels.

		**Direction of effect**		***P*-value**
***S. mutans***				
	TSBS		arginine 10 mg/ml	0.2437
	TSBS	<	nicotine 4 mg/ml	0.0007
	TSBS		arginine 10 mg/ml + nicotine 4 mg/ml	0.1748
	arginine 10 mg/ml	<	nicotine 4 mg/ml	0.0202
	arginine 10 mg/ml		arginine 10 mg/ml + nicotine 4 mg/ml	0.8213
	nicotine 4 mg/ml	>	arginine 10 mg/ml + nicotine 4 mg/ml	0.0404
**EPS**				
	TSBS		arginine 10 mg/ml	0.1374
	TSBS		nicotine 4 mg/ml	0.3669
	TSBS	>	arginine 10 mg/ml + nicotine 4 mg/ml	0.0061
	arginine 10 mg/ml	<	nicotine 4 mg/ml	0.0227
	arginine 10 mg/ml		arginine 10 mg/ml + nicotine 4 mg/ml	0.1783
	nicotine 4 mg/ml	>	arginine 10 mg/ml + nicotine 4 mg/ml	0.0006
**EPS:** ***S. mutans***				
	TSBS	>	arginine 10 mg/ml	0.0022
	TSBS	>	nicotine 4 mg/ml	0.0332
	TSBS	>	arginine 10 mg/ml + nicotine 4 mg/ml	0.0001
	arginine 10 mg/ml		nicotine 4 mg/ml	0.3356
	arginine 10 mg/ml		arginine 10 mg/ml + nicotine 4 mg/ml	0.3469
	nicotine 4 mg/ml		arginine 10 mg/ml + nicotine 4 mg/ml	0.0649

## Discussion

Previous studies report that compared to serotype *c* strains of *S. mutans*, serotype *k* strains produce less glucose-induced acid, and demonstrate low levels of sucrose-dependent adhesion and dextran-binding activity [4, 19]. Additionally, serotype *k* strains are less susceptible to phagocytosis by human polymorphonuclear leukocytes which may explain the increased prevalence of serotype *k* strains isolated from infected mammalian soft tissues [19]. We report a large survey of the biofilm growth characteristics of *S. mutans* serotype *k* strains in comparison to *S. mutans* serotype c strains. There was an insignificant increase in biomass of *S. mutans* serotype *c* strains compared to serotype *k* strains when grown in TSBS alone. In contrast, the metabolic activity of the serotype *k* strains was increased compared to the serotype *c* strains studied but this also was not significant. CLSM analyses of 3 strains of each serotype did not reveal statistical differences between the serotypes for volume of either bacterial cells or EPS. These observations expand on an earlier study of serotype *k* strains NUM-Smk-51, NUM-Smk52 and NUM-Smk89, wherein sucrose induced glucan synthesis and plaque formation by these three serotype *k* strains were similar to serotype *c* strains [4]. Yamamoto and Takada reported these serotype *k* strains had lower acid production in the presence of glucose compared to serotype *c* strains which may relate to the theory that serotype *k* strains are less cariogenic than serotype *c* strains. The serotype of the *S. mutans* strains used in this study did not have a significant effect on sucrose-induced biofilm growth.

Thirty of the strains were previously genotyped for CBP genes allowing analysis of the effects of the *cbm* and *cnm* genes on development of sucrose induced biofilm. The CBP genotype of the strain had significant effects on biofilm production. When grown in TSBS alone, *cbm*-/*cnm*+ strains produced significantly more biofilm and metabolic activity was increased compared to *cbm*-/*cnm*- strains. The *cbm*+/*cnm*- strains produced moderately increased biofilm but the metabolic activity of these strains was significantly higher than found for the strains lacking both genes. While *cbm*+/*cnm*- and *cbm*-/*cnm*+ strains are not statistically different from each other, there does not appear to be a synergistic effect of *cbm* and *cnm* genes on biofilm production as both biomass and metabolic activity was decreased for strains bearing both genes (*cbm*+/*cnm*+) compared to strains possessing only one of the CBP genes. From our data, the biomass and metabolic activity of *cbm*+/*cnm*+ strains were not statistically different from the strains lacking both genes. In the CLSM studies the *cbm*-/*cnm*+ strain produced significantly more EPS than the *cbm*+/*cnm*- and *cbm*-/*cnm*- strains. Abranches et. al. investigated 5 *cnm*+ *S. mutans* strains and the respective *cnm* knockout strains in biofilm grown on saliva coated microtiter wells in media supplemented with either 1% sucrose and 1% glucose [7]. In these studies, no difference in biomass was observed for 4 of the 5 knockout pairs in 1% sucrose and 3 of 5 knockout pairs were not statistically different grown in 1% glucose. These investigators concluded biofilm formation was strain specific and the *cnm* gene did not contribute to biofilm formation. Our studies of clinical isolates confirm strain to strain variation in biofilm production even among strains with the same CBP genotype. Additional studies are warranted to determine whether *cnm* knockout strains are comparable to native *cbm*-/*cnm*- clinical isolates.

The addition of 4 mg/ml nicotine to TSBS significantly increased both the biomass and the metabolic activity of *S. mutans* biofilms. This is the first report of the effects of nicotine on serotype *k S. mutans* and we report no significant differences in biomass, metabolic activity or EPS production compared to serotype *c* strains upon exposure to nicotine. Our findings are consistent with previous studies using *S. mutans* serotypes *c, e* and f wherein *S. mutans* biofilm growth and metabolism were increased in a nicotine dose dependent manner [10, 11, 20]. In the present study nicotine was added at the beginning of biofilm formation whereas the 2012 study by Huang et al. added nicotine to established 24 h *S. mutans* biofilms and the metabolic activity was measured 24 h later. Given that similar nicotine dose response profiles were reported for nascent and established biofilm, comparatively these studies indicate that nicotine increases *S. mutans* biofilm growth regardless of when nicotine exposure occurs.

The CLSM studies with six *S. mutans* strains revealed that 4 mg/ml nicotine increased *S. mutans* bacterial volume which correlates with the increase in metabolic activity in the presence of 4 mg/ml nicotine. Our findings are supported by previous studies of *S. mutans* UA159 (serotype *c*) biofilm which reported increased bacterial volume with nicotine doses of 2 and 4 mg/ml in single- and dual-species biofilm [11, 21]. In studies of cigarette smoke exposure, another *S. mutans* strain (ATCC 25175) displayed increased colony size as the nicotine content of the cigarette increased [22]. Zonuz et al. reported a 51% greater increase in *S. mutans* colony size compared to *Streptococcus sanguinis*. In our study of 6 strains, nicotine did not have a significant effect on EPS production whereas other single- and dual-species biofilm studies found increased EPS production in the presence of nicotine. Single-species studies of sucrose-induced UA159 biofilm found the EPS volume was significantly increased by addition of 2 and 4 mg/ml nicotine [11]. In dual-species biofilm with *S. sanguinis*, EPS production by UA159 in 2 mg/ml nicotine was significantly increased compared to biofilm grown without nicotine [21]. In both studies, only UA159 was investigated. Our data suggest that increased EPS production in the presence of nicotine may be a characteristic of UA159 and may not be attributable to all strains of *S. mutans*.

Overall, addition of 10 mg/ml arginine to sucrose-induced biofilm had no effect on the metabolic activity of *S. mutans* biofilm. Collectively, the biomass was unchanged by addition of 10 mg/ml arginine for the strains investigated. In contrast, *cbm*+/*cnm*- strains produced significantly less biomass in the presence of 10 mg/ml arginine compared to TSBS alone. Significantly more biomass was produced by *cbm*-/*cnm*+ strains than *cbm*+/*cnm*- and *cbm*-/*cnm*- genotypes in the presence of arginine. The metabolic activities of the *cbm*-/*cnm*+ strains were greater than observed for strains lacking both genes but were not statistically different from *cbm*+/*cnm*- strains. CLSM studies demonstrated that strains with a CBP gene treated with arginine had greater proliferation. In contrast, EPS production was decreased by addition of arginine. There was no significant change in either *S. mutans* or EPS volume in the presence of arginine, however, the EPS:*S. mutans* bacteria ratios were significantly lower when compared to biofilm grown in TSBS alone indicating a decrease in EPS relative to *S. mutans* volume. These data are consistent with previous studies of single type strains of *S. mutans* which have shown that addition of arginine results in increased biomass with decreased EPS production (UA159) and a more fragile sucrose-induced *S. mutans* biofilm (UA140) compared to biofilm grown in the absence of arginine [12, 16, 23, 24]. Using two different nutrient-rich media, Chakraborty and Burne demonstrated that 1.5% arginine induced decreases in overall biofilm production and differences in UA159 growth curves [17]. The relative amount of L-arginine and the growth media used in these experiments vary thereby demonstrating that the effect of arginine on *S. mutans* biofilm is not dependent upon the specific growth media and concentration as the effects were observed over a range of arginine concentrations (0.5–10%). In each of the aforementioned investigations arginine was present at the beginning of biofilm formation. There is no evidence to suggest that arginine affects established biofilm, however, one can speculate that biomass and EPS production after addition of arginine would replicate the nascent arginine-grown biofilm.

Nicotine upregulates *S. mutans* UA159 virulence genes, glucosyltransferases, glucan binding proteins and bacteriocin production [11, 25, 26] (Shepherd et al., unpublished). *S. mutans* antigen I/II was upregulated in strain NG8 biofilm in the presence of nicotine [20]. In contrast, Chakraborty and Burne demonstrated that arginine downregulates genes encoding virulence factors responsible for attachment, competence development and bacteriocin production [17]. *S. mutans* displayed lower tolerance to environmental acid and oxidative stress in the presence of arginine [17]. Because nicotine and arginine exert opposite effects on *S. mutans* UA159, we investigated the combined effects on biofilm production by UA159 and 32 additional strains of *S. mutans*. Our results support published observations that nicotine enhanced biofilm production and arginine decreased biofilm. Furthermore, when combined, arginine and nicotine enhanced biomass and metabolic activities compared to nicotine alone as shown by the crystal violet and XTT experiments. The CLSM experiments with a limited number of strains demonstrated that addition of arginine to nicotine resulted in significantly decreased volume of *S. mutans* for the *cbm*+/*cnm*- strains and decreased EPS volume for all strains compared to biofilm grown with nicotine alone. These data indicate that arginine diminishes nicotine enhanced EPS production. The nicotine enhanced *S. mutans* bacterial growth of strains bearing the *cbm* gene was reduced by adding arginine to nicotine. Additional studies are required to determine the mechanism(s) by which arginine reduces nicotine increased EPS production, however, one might speculate that arginine acts upon regulatory processes or upstream of the point of nicotine interaction with genes that control EPS production.

Two clinical studies found that tobacco smokers do not have increased *S. mutans* in saliva compared to non-smokers [27, 28]. These studies did not type for CBP in oral isolates. We found nicotine enhanced biofilm production for all strains. The biofilm metabolic activities for strains bearing *cbm* and *cnm* genes were increased compared to strains without genes for CBP. Previous studies have shown that the *cnm* gene contributes to oral colonization by *S. mutans* and is a predictor of development and severity of dental carious lesions [29–31]. The *cbm* gene has 78% identity with the *cnm* gene and exhibits greater collagen binding activity than the *cnm* gene [32]. The relationship between the two genes is controversial; some investigations report the genes to be located within the same locus whereas others report multiple strains bearing both genes simultaneously [8, 33]. Our data from multiple strains indicate similarities between *cbm* and *cnm* in biomass and metabolic activities for strains with either gene. Strains bearing both genes do not demonstrate synergistic effects and were found to have lower responses to nicotine than strains with either gene alone. These observations suggest *cbm* and *cnm* are separate genes with significant sequence identity. Full genetic sequencing of these strains may lead to better understanding of the differences between strains beyond the CBP genes and can identify additional genetic variations between strains which can affect metabolic activity and biomass growth.

In conclusion, serotype *c* and serotype *k* strains are not significantly different in terms of biofilm production or responses to nicotine and arginine. Nicotine increased biomass and metabolic activity in the strains studied. The presence of *cbm* and *cnm* affected biofilm growth and responses to nicotine such that strains with a CBP produced more biomass and were more metabolically active. Addition of nicotine resulted in increased biofilm for all strains. Arginine added with nicotine reduced the quantity of bacteria and EPS in the biofilm. Inasmuch as arginine has been added to fluoride in dentifrices to enhance the anti caries effect of fluoride, this combination may be especially beneficial for tobacco users due to the ability of arginine to reduce nicotine-induced EPS production.

## Data Availability Statement

The raw data supporting the conclusions of this article will be made available by the authors, without undue reservation.

## Author Contributions

DW and RG participated in conception of the work. DW designed and executed the experiments and analyzed the data and drafted the manuscript. RG critically reviewed and edited the manuscript. Both authors contributed to the article and approved the submitted version.

## Funding

This study was supported in part by the Indiana University School of Dentistry Ph.D. Student Research Fund and Franciscan Health Indianapolis.

## Conflict of Interest

The authors declare that the research was conducted in the absence of any commercial or financial relationships that could be construed as a potential conflict of interest.

## Publisher's Note

All claims expressed in this article are solely those of the authors and do not necessarily represent those of their affiliated organizations, or those of the publisher, the editors and the reviewers. Any product that may be evaluated in this article, or claim that may be made by its manufacturer, is not guaranteed or endorsed by the publisher.
