# Supplementary material for: Analyses of the Effects of Arginine, Nicotine, Serotype and Collagen-Binding Proteins on Biofilm Development by 33 Strains of Streptococcus mutans
Source: Front Oral Health. 2021 Nov 25;2:764784. doi: 10.3389/froh.2021.764784 (PMC8757754; doi:10.3389/froh.2021.764784)
Supplement: Supplementary file 1 [file Data_Sheet_1.PDF]

Supplementary Material

1 Supplementary Data

1.1 Nicotine and arginine titration

Working under the observation that serotype *k* strains of *S. mutans* differ from serotype *c* strains in terms of prevalence in the oral cavity and in association with extra-oral tissue infection, we selected 10 strains of each serotype to determine whether the serotype influenced biofilm growth. In previous studies of serotype *c*, *e* and *f* strains, nicotine enhanced *S. mutans* biofilm growth. Nicotine dose titration curves of the 10 selected serotype *k* (Figure 1, panel A) and serotype *c* (Figure 1, panel B) strains were consistent across the strains. As measured by crystal violet, the biofilm mass of each strain peaked at either 4 or 8 mg/ml nicotine. Universally, no biofilm growth was observed with 16 mg/ml nicotine. Based upon these experiments, doses of 0 and 4 mg/ml of nicotine were selected for the present study of biofilm growth.

In order to determine the arginine concentration for subsequent studies, a dose titration was performed for the same 10 strains (Figure 2). There was no difference in biofilm mass between 0 and 5 mg/ml arginine doses measured by crystal violet staining (p=0.9325, Figure 2A serotype *k*, 2B serotype *c*). Compared to biofilm grown without arginine, increases in biofilm mass occurred in the presence of 10 and 20 mg/ml arginine (p=0.0339 & 0.0253, respectively). Biofilm grown in 10 mg/ml arginine was not different from 20 mg/ml arginine (p=0.9078). Doses of 40 and 80 mg/ml arginine resulted in significant decreases in biofilm mass compared to baseline (p<0.001). Similarly, bacterial metabolism measured by the XTT assay was increased by doses of 10 and 20 mg/ml arginine (p=0.0133 and 0.0054, respectively) and decreased by 40 and 80 mg/ml doses (p=0.0016 and <0.0000; Figure 2C serotype *k*, 2D serotype *c*). When analyzed individually, several strains displayed lower biofilm mass and metabolism with 20 mg/ml than with 10 mg/ml arginine. Collectively, these observations lead us to select 10 mg/ml arginine for subsequent experiments.

2 Supplementary Figures and Tables

2.1 Supplementary Figures

Figure S1.

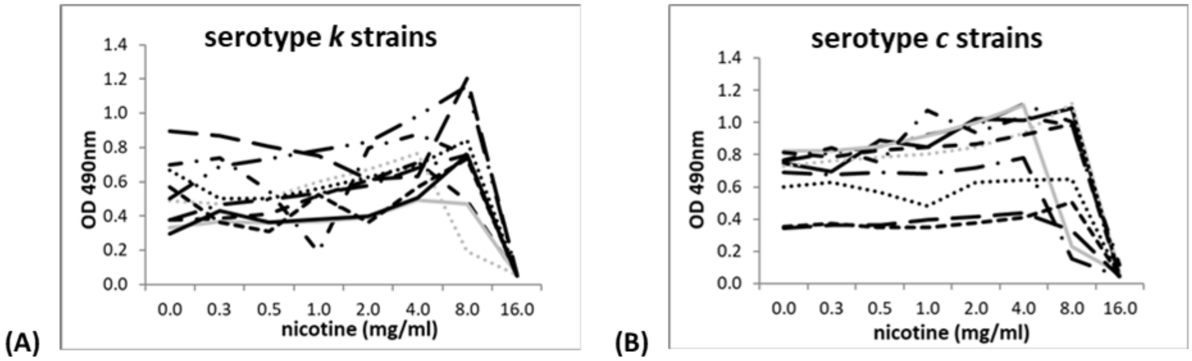

Supplementary Figure 1. Effects of nicotine on *S. mutans* biofilm formation. Nicotine was added to

TSBS in doubling doses from 0.25 to 16 mg/ml prior to addition of *S. mutans* into quadruplicate wells of a 96-well plate and incubation at 37ºC, 5% CO_2 for 24 h. The 24 h biofilm from 10 serotype *k* (A) and 10 serotype *c* (B) strains were stained with crystal violet. The mean absorbance for each dose is shown.

Figure S2.

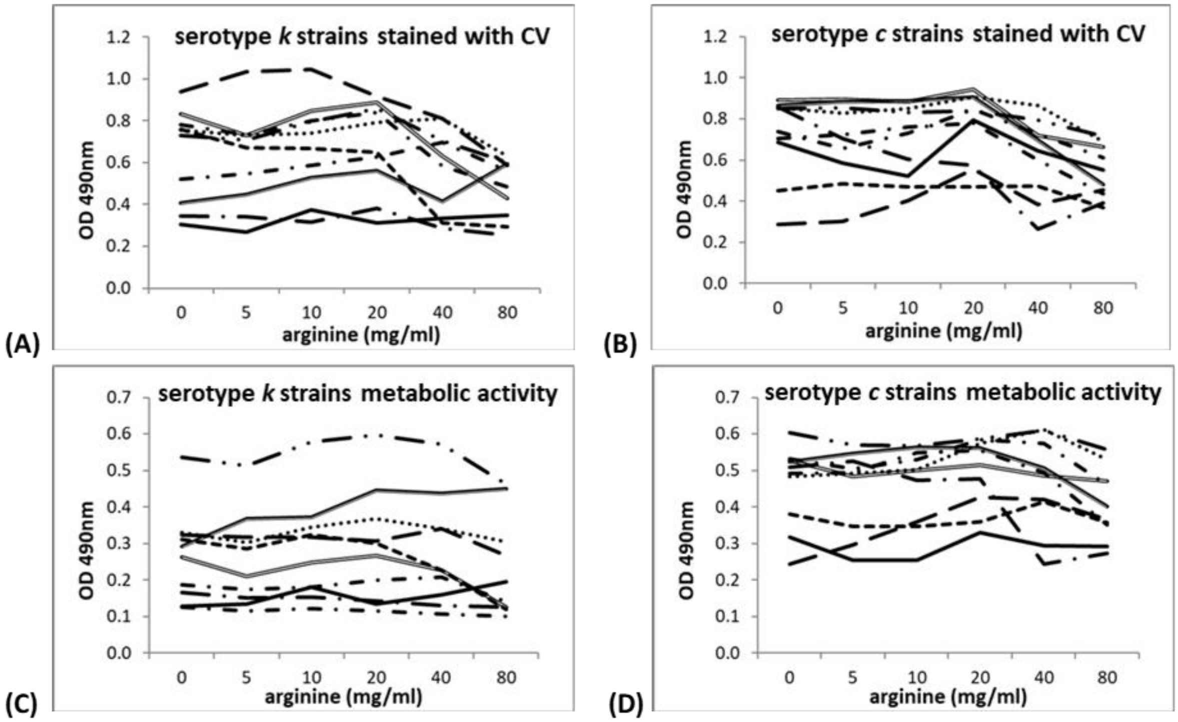

Supplementary Figure 2. Selected strains of *S. mutans* serotypes k and c biofilm in studies of dose response to arginine. Biomass of serotype *k* (A) and *c* (B) strains stained with crystal violet. Metabolic activity of serotype *k* (C) and *c* (D) strains as measured by the XTT assay.

Table S1. The effects of *S. mutans* serotype on biofilm responses to arginine (10 mg/ml) and nicotine (4 mg/ml)

		S. mutans serotype (n)				
		c (10)		*k (10)*		Serotype *c* vs *k*
		OD 490nm		OD 490nm		
Arginine (10 mg/ml)	Nicotine (4 mg/ml)	mean	SD	mean	SD	P value
**Crystal Violet – Biofilm Mass**						
-	-	0.717	0.217	0.671	0.225	0.199
+	-	0.689	0.198	0.628	0.209	0.113
-	+	0.938	0.308	1.019	0.397	0.634
+	+	0.930	0.363	1.066	0.463	0.188
**XTT – Biofilm Metabolic Activity**						
-	-	0.461	0.144	0.518	0.291	0.248
+	-	0.464	0.131	0.508	0.297	0.284
-	+	0.542	0.159	0.657	0.372	0.231
+	+	0.573	0.170	0.680	0.391	0.527
